# The remote flap technique: recruiting regional laxity for reconstruction of high-tension cutaneous defects a descriptive single-surgeon case series

**DOI:** 10.1016/j.jpra.2026.08.002

**Published:** 2026-08-11

**Authors:** Ryan James Livingston

**Affiliations:** a Director of Plastic Surgery, Sunshine Coast University Hospital, Sunshine Coast, Queensland, Australia; b Griffith University, Gold Coast, Queensland, Australia; c Kawana Private Hospital, Sunshine Coast, Queensland, Australia

**Keywords:** Remote flap, Regional laxity, Reconstructive surgery, Local flap, Skin cancer, High-tension defect

## Abstract

**Background:**

Local flaps recruit laxity into a defect through incisions that extend from the defect into the immediately surrounding tissue; these incisions can disturb peri-defect perfusion and place scars within the aesthetic unit of the defect. The remote flap technique is a reconstructive strategy rather than a single flap entity. A zone of regional or distant laxity is identified away from the defect and recruited to achieve closure without an incision connecting the flap to the defect — the feature that distinguishes it from a conventional local flap — keeping the reconstructive work, and the resulting scar, away from the defect where this is advantageous. The flap raised at the remote site may itself be constructed as a local, pedicled, or free flap.

**Methods:**

A retrospective audit of 100 consecutive patients (112 remote flaps) treated at Kawana Private Hospital, Sunshine Coast, Australia, between 25 May 2022 and 1 January 2025 was performed, and flap-related complications were recorded. The operative principles, flap design, and a decision framework for the technique are described, with selected additional cases presented to illustrate specific considerations.

**Results:**

All 100 patients achieved successful reconstruction with 112 remote flaps. Lesions were squamous cell carcinoma (46.4%), basal cell carcinoma (26.8%), invasive melanoma (8.9%), melanoma in situ (8.0%), intraepidermal carcinoma (8.9%), and other (0.9%). Flap-related complications were infrequent: infection 3.6% (4/112) and dehiscence 0.9% (1/112), total 4.5% (95% CI 1.5–10.1%). Excision-related incomplete margins, independent of the flap, occurred in 1.8% (2/112).

**Conclusion:**

In this single-surgeon series the remote flap technique was a safe and reproducible option for high-tension cutaneous defects, with a flap-related complication profile consistent with published local-flap reconstruction. Potential advantages in local tissue preservation and scar placement are described but were not formally measured and require prospective comparative study.

## Introduction

Reconstructive surgery traditionally defines the defect, plans in reverse, and recruits laxity to achieve closure, most often with a local flap such as an advancement, rotation, or transposition.^1^, ^2^, ^3^, ^4^ Because a local flap borrows tissue immediately adjacent to the defect, its incisions may reduce perfusion in the peri-defect tissue and concentrate scarring within the same aesthetic unit, occasionally contributing to wound-edge breakdown and conspicuous scars.^5^, ^6^, ^7^, ^8^

The remote flap technique instead recruits laxity from a site remote from the defect. Its defining feature — and what separates it from a conventional local flap — is that no incision connects the flap to the wound. A local flap borrows adjacent tissue through an incision extending from the defect; a remote flap is raised at a distant point of maximum laxity, oriented away from the defect, and the recruited laxity is transmitted across the intervening tissue to allow closure. It is therefore a strategy rather than a single named flap, and it is not a distant relaxing incision. The flap raised at the remote site may itself be constructed as a local, pedicled, or free flap; the distinguishing principle lies in recruiting distant laxity without a bridging incision, rather than in the flap used to do so.

The mechanism is most easily understood as a chain: the defect is one link, the intervening tissue forms the middle links, and the remote flap is a distant link. Moving the distant link releases tension along the whole chain and transmits laxity toward the defect.^9^, permitting closure without an incision joining the two.

The point of maximum laxity serves as a common decision point in planning. A short incision made here allows the available laxity to be assessed directly and, in some cases, closed perpendicularly to recruit tissue toward the defect (closing a width x yields approximately x/2 of advancement), which may achieve closure without any flap. Where more tissue is required, this incision can either be continued toward the defect — producing a conventional local flap — or used to raise a flap directed away from the defect: the remote flap, with no incision joining it to the wound. The remote flap is chosen when it recruits superior laxity or relocates the donor scar to a less conspicuous site, and when avoiding a bridging incision spares further scarring within the aesthetic unit of the defect. The principles of moving tissue on its own vascular basis are shared with perforator, propeller, and keystone flaps.^10^, ^11^, ^12^, ^13^; the remote flap is distinguished by recruiting regional laxity from a discontinuous donor site, without an incision connecting flap to defect.

This paper describes the technique and its design rationale, supported by Figs. (Figs. 1–8), presents an audit of 112 remote flaps in 100 consecutive patients, and discusses selected cases that illustrate specific clinical considerations.

**Fig. 1:**
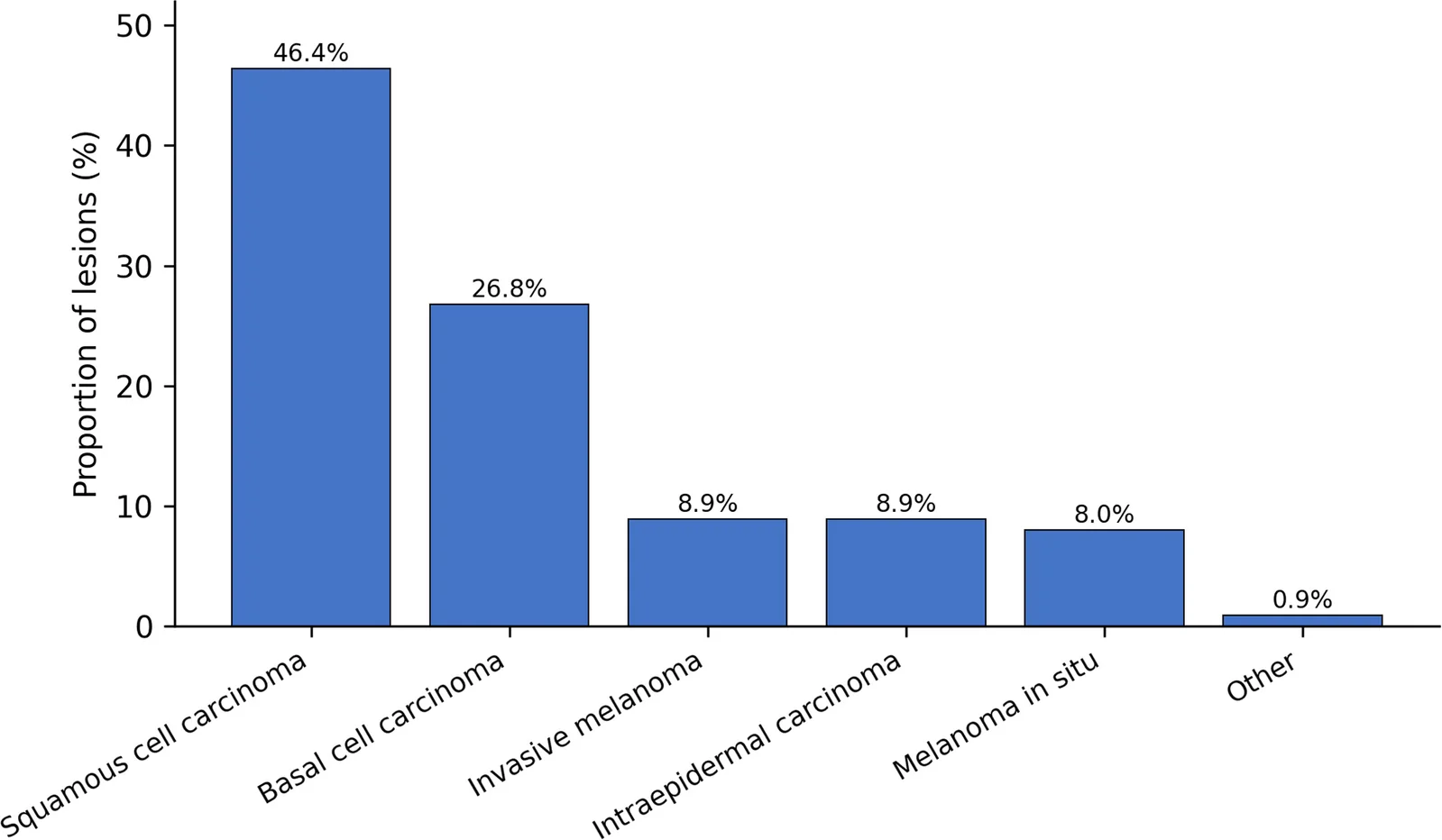
Bar chart of lesions by skin cancer type (n = 112).

**Fig. 2:**
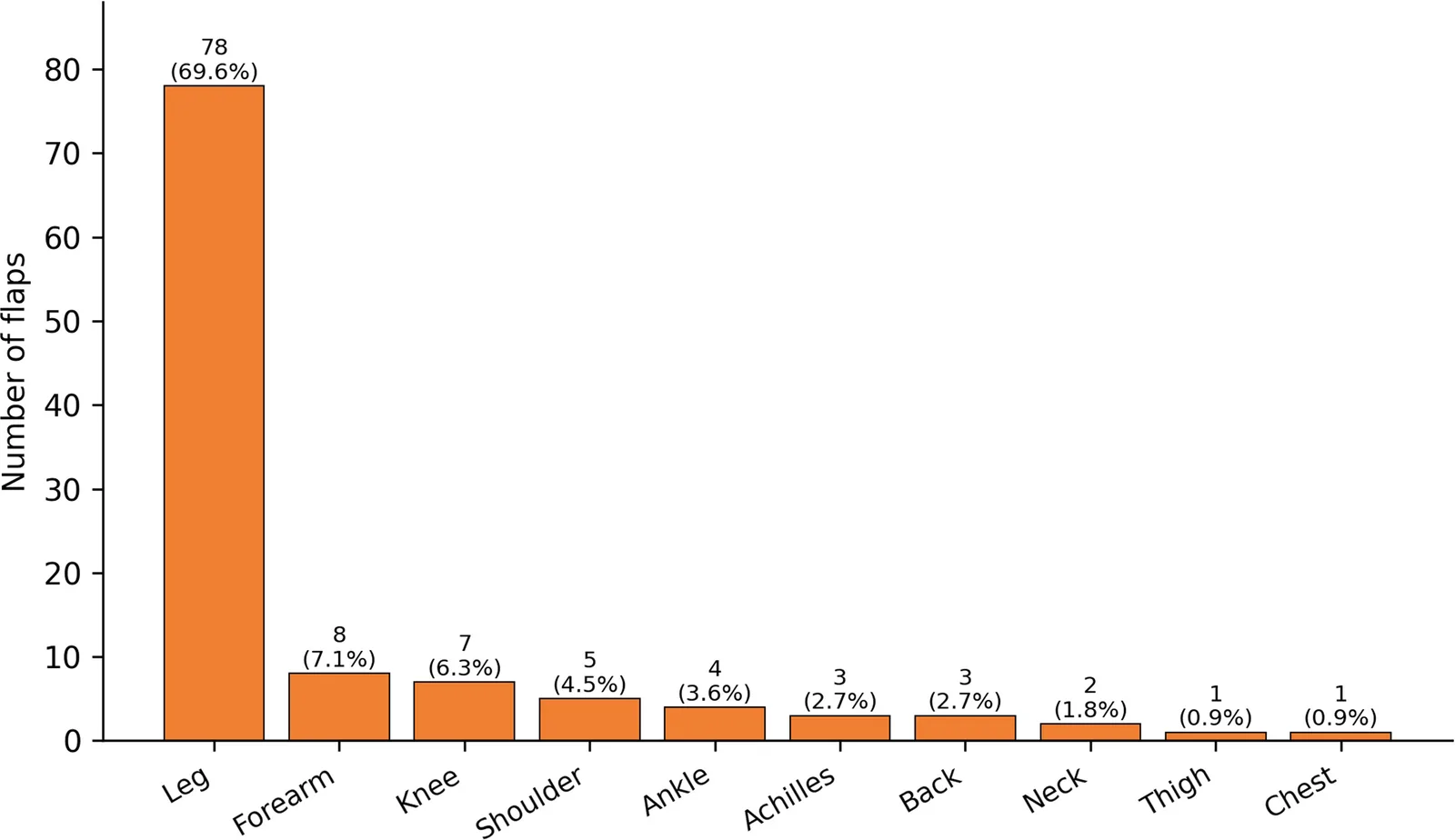
Bar chart of remote flaps by anatomical site (n = 112).

**Fig. 3:**
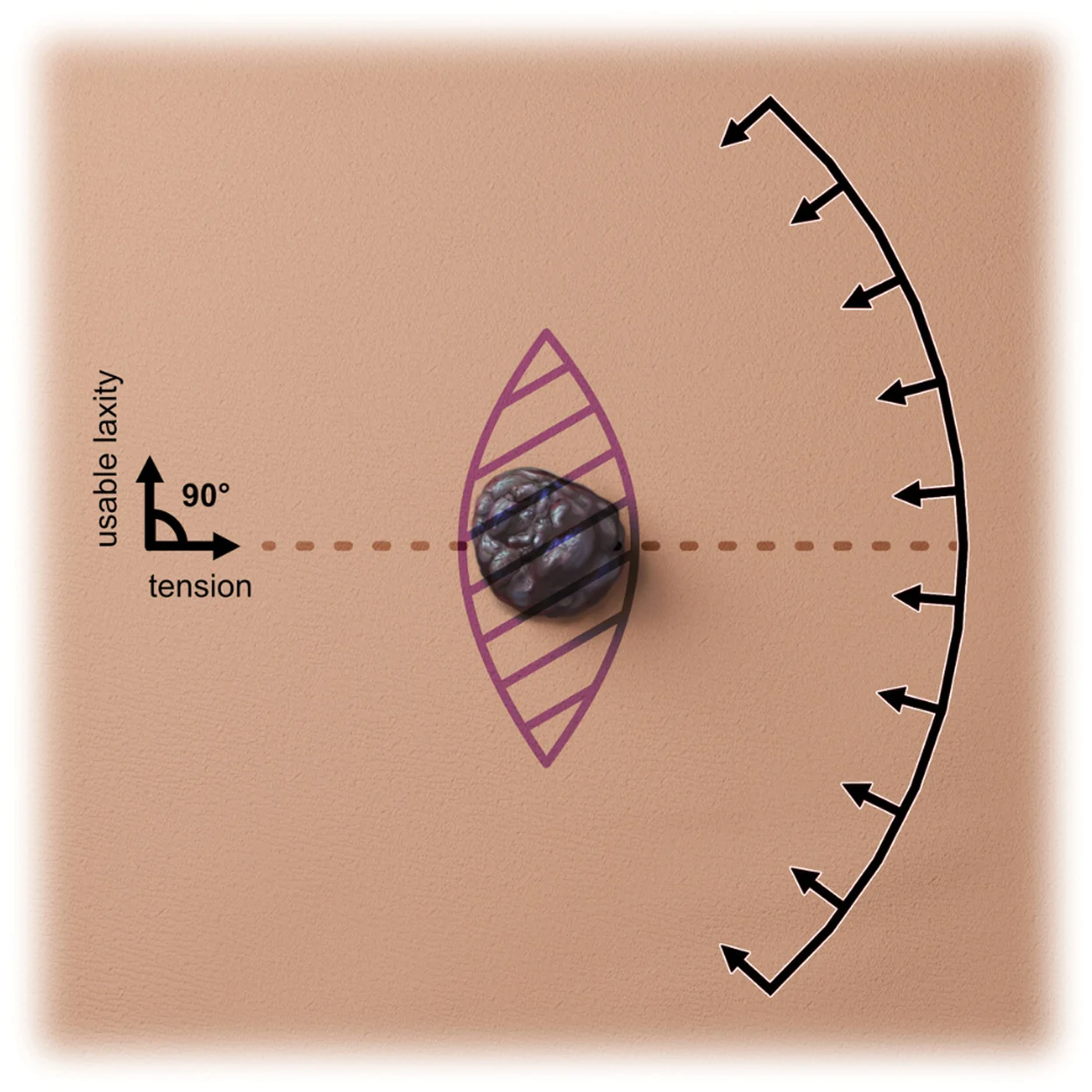
Usable skin laxity, often oriented at approximately 90° to the defect’s principal tension vector, available for recruitment toward the defect during flap design.

**Fig. 4:**
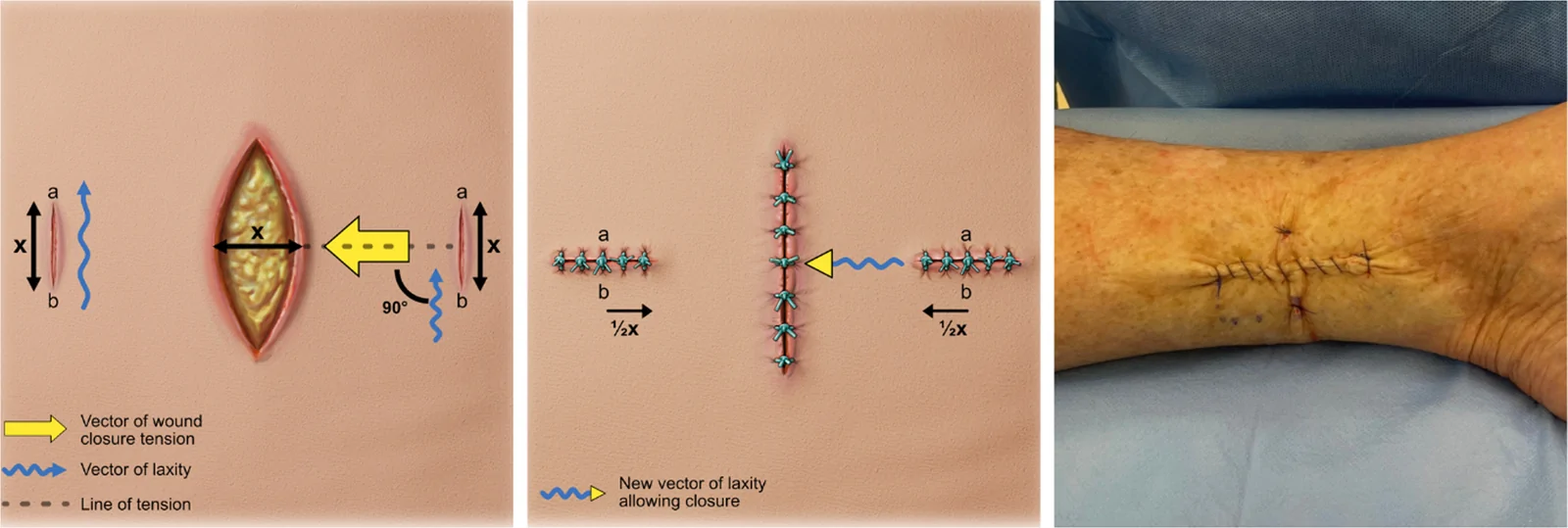
A laxity zone closed perpendicularly to recruit tissue toward the wound via the “chain effect”: closing a width x recruits approximately x/2 of advancement toward the defect. This closure is performed at a common point on either side of the defect to allow closure.

**Fig. 5:**
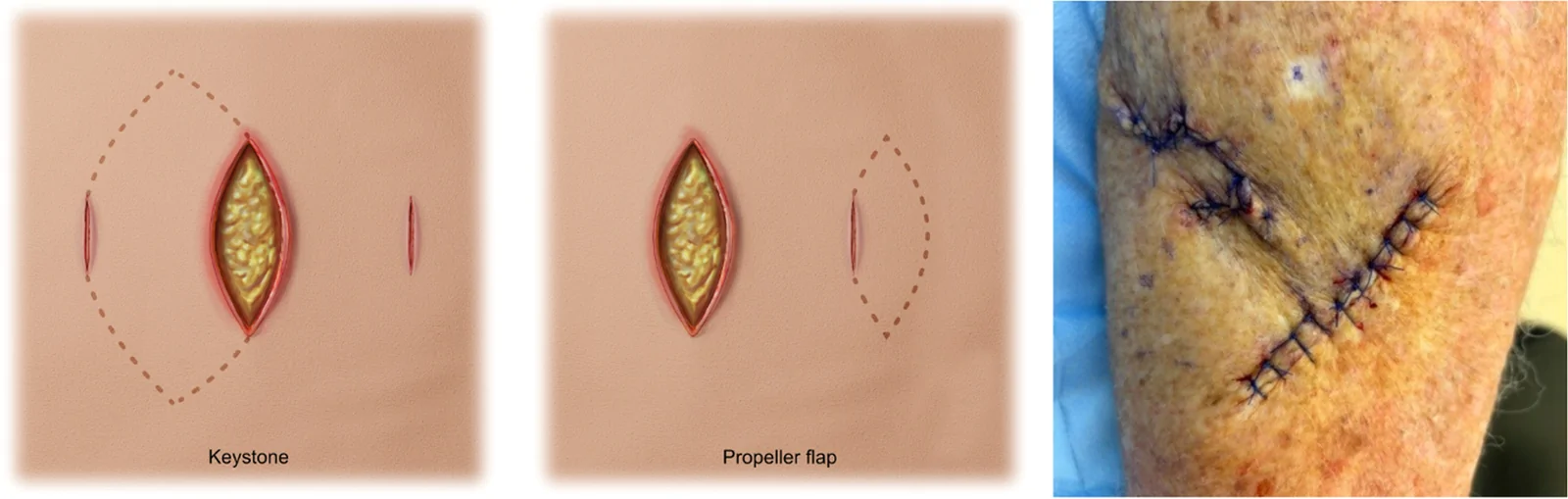
A common point (often in the same or an adjacent angiosome) with laxity that can be closed perpendicularly, recruited, or converted to a remote or local flap, transmitting laxity and enabling closure. The illustration shows conversion into a keystone or propeller flap; in practice a range of options is available (advancement, rhombic, hatchet). The photograph shows a case where the common point was converted into a remote transposition flap to promote closure.

**Fig. 6:**
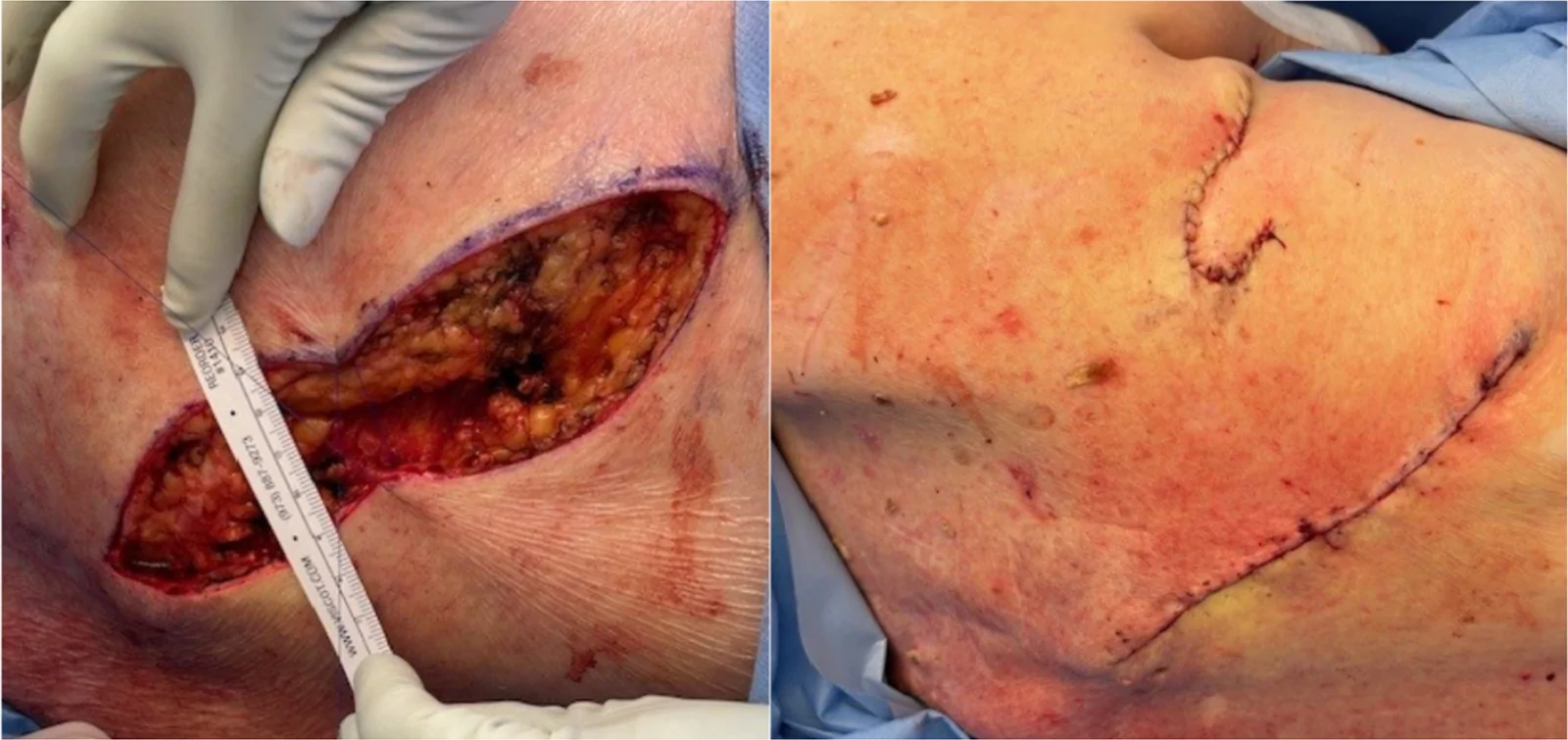
A large back lesion with a remote flap raised in a zone of laxity in the flank to promote closure. The first photograph shows attempted closure after undermining and skin-adhesion release; the second shows successful closure using a remote transposition flap based in the flank.

**Fig. 7:**
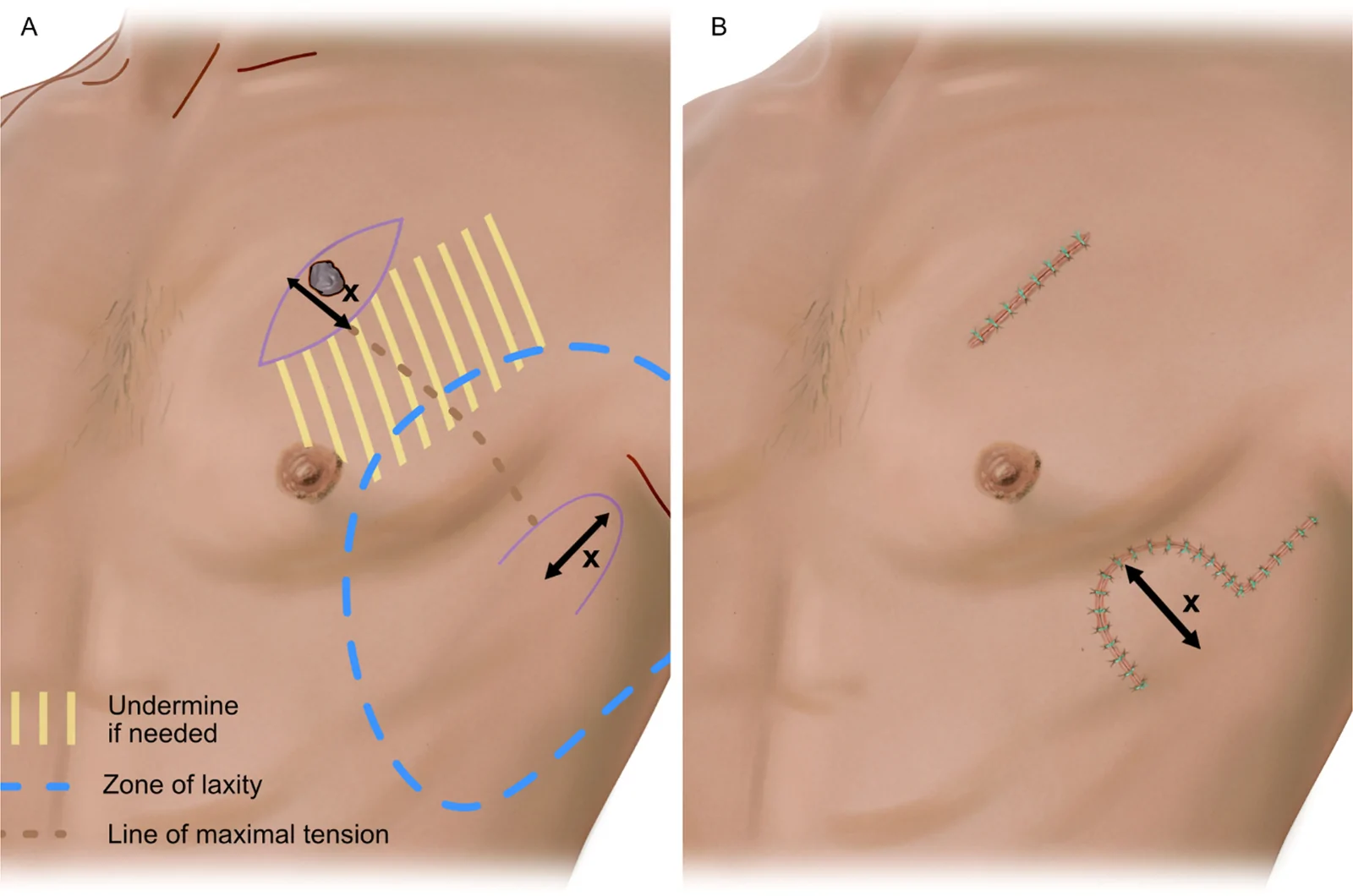
The technique used to repair an excision defect overlying a pacemaker. (A) The zone of laxity used by the remote flap and the surgically undermined area released to allow tension-free closure. (B) The offset suture line. An actual case performed by the author.

**Fig. 8:**
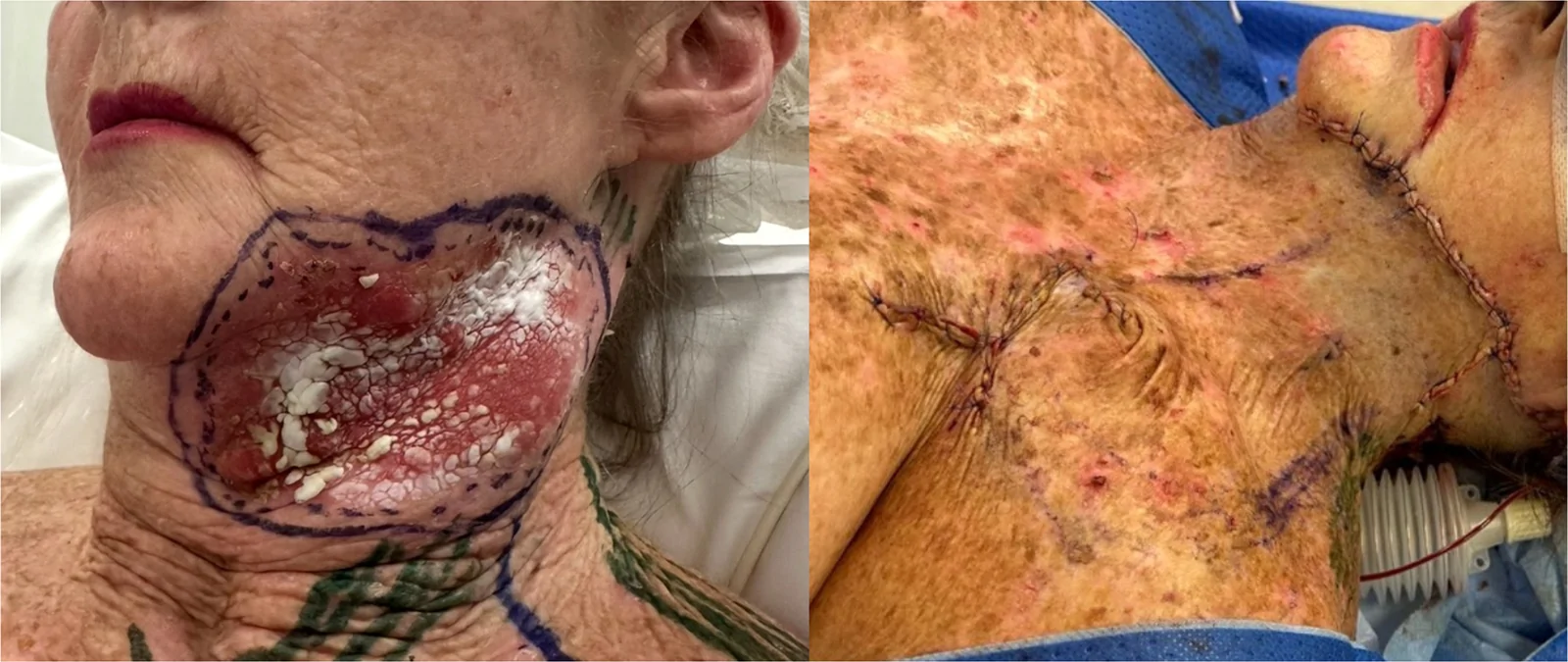
A large cheek defect closed with undermining and a remote flap based on the left chest, with no scar placed on the neck as per the patient’s request.

## Methods

A retrospective audit was performed at Kawana Private Hospital, Sunshine Coast, Queensland, of 100 consecutive patients who underwent remote flap procedures between 25 May 2022 and 1 January 2025, totalling 112 flaps; some patients required more than one flap. Appropriate approvals and consents were obtained, including permission from Kawana Private Hospital and the data custodian. De-identified data recorded in Microsoft Excel included demographics, lesion type, size, location, and outcomes. Lesion diameter (shortest axis plus margins) was verified by independent photographic-ruler assessment immediately before surgery. Flaps targeted high-tension sites (e.g., lower limb, joints) unsuitable for direct closure, with sizes of 1.5 to 4.5 cm (mean 2.3 ± 0.8 cm). Lesion types were squamous cell carcinoma (46.4%), basal cell carcinoma (26.8%), invasive melanoma (8.9%), melanoma in situ (8.0%), intraepidermal carcinoma/squamous cell carcinoma in situ (8.9%), and other (0.9%). Sites were leg (69.6%), forearm (7.1%), knee (6.3%), shoulder (4.5%), ankle (3.6%), Achilles (2.7%), back (2.7%), neck (1.8%), thigh (0.9%), and chest (0.9%).

Data captured site, size, infection, dehiscence, incomplete margins (excision-related), and fragile suture lines (defined as cases requiring suture retention 3–5 days beyond the planned 7 days). Complications were benchmarked against published local-flap and reconstruction literature.^15^^,^^16^ Statistical analysis was descriptive, with 95% confidence intervals calculated by the Clopper–Pearson method.^18^ Two additional cases — a 6 cm cheek defect and a cancer overlying a pacemaker — are presented to illustrate specific considerations; patient consent for images and publication was obtained.

### Operative technique and flap design

The technique proceeds through a reproducible sequence. (i) Assess the defect: determine its size, the principal tension vector, and the orientation of adjacent relaxed skin tension lines (RSTLs). (ii) Map regional laxity: using a manual pinch test in several directions, identify the point of maximum recruitable laxity in the region; laxity is frequently maximal at approximately 90° to the defect's tension vector (Fig. 3). (iii) Evaluate the intervening tissue: note anatomical adhesion zones (e.g., over retinacula, joints, or fascial attachments) and the vascular territory (angiosome) between the laxity point and the defect, as both influence how efficiently laxity is transmitted.^7^^,^^8^ (iv) Incise and test the common point: a short incision at the common point (Fig. 5) allows laxity to be assessed directly and, if sufficient, closed perpendicularly — closing a width x recruits approximately x/2 of advancement toward the defect (Fig. 4) — which in some cases achieves closure without any flap. (v) If more tissue is needed, choose between a conventional local flap (continuing the incision toward the defect) and a remote flap (raising the flap away from the defect, with no incision connecting it to the wound); the flap raised may be constructed as a local, pedicled, or free flap as dictated by the defect and donor site. When the flap is raised away from the wound, it is designed so that the additional laxity it liberates is directed maximally toward the incision site — and thence along the chain toward the defect — by aligning the flap's orientation, and the vector of its recruited slack, with the direction of required closure.

**Choosing between perpendicular closure, a local flap, and a remote flap.** Perpendicular closure of the common point alone is used when the recruited laxity is sufficient to close the defect. A conventional local flap is chosen when continuing the incision toward the defect provides adequate tissue with an acceptable scar. A remote flap is chosen when raising the flap away from the defect recruits greater or more reliable laxity, or places the donor scar where it is better concealed, and when avoiding a bridging incision spares the peri-defect tissue and its aesthetic unit. Where two options are otherwise equivalent, the one that avoids releasing anatomical adhesions is preferred, unless release yields a clear functional or aesthetic gain.

**Distance and adhesions.** In the author's experience, the efficiency with which laxity reaches the defect falls as the distance from the remote flap to the defect increases, and this is compounded by intervening adhesions, which act as fixed anchors along the chain. Distance is therefore assessed intra-operatively by direct laxity transmission (the common-point test) rather than by a fixed measurement, since the same absolute distance transmits laxity differently depending on the adhesions and tissue mobility between the two points. Where safe and beneficial, anchoring adhesions are released to permit transmission of laxity; where a design can avoid them, that design is generally favoured.

**Scar orientation and RSTLs.** Donor scars are planned, where possible, to lie within or parallel to RSTLs or at aesthetic-unit boundaries. In some designs the remote scar is deliberately placed across an RSTL when this is the price of recruiting a superior reservoir of laxity or of relocating the scar to a more concealable site; this is a considered trade-off recorded during planning rather than an oversight.

**Relative contraindications.** These include poor vascularity at the remote site, previous radiotherapy of the donor region, extensive intervening adhesion zones that cannot be safely released, and any defect better served by another rung of the reconstructive ladder.

## Results

All 100 patients achieved successful reconstruction with 112 remote flaps, with 100% flap survival and no scar revisions at the time of writing. Table 1 summarises outcomes. Flap-related complications comprised 4 infections (3.6%; 95% CI 1.0–8.9%) and 1 dehiscence (0.9%; 95% CI 0.0–4.9%), totalling 4.5% (95% CI 1.5–10.1%). Excision-related incomplete margins, independent of the flap, occurred in 1.8% (2/112; 95% CI 0.2–6.3%). Eight flaps (7.1%) had fragile suture lines requiring delayed suture removal. By site, the leg (78 flaps, 69.6%) accounted for 2 infections and 1 minor dehiscence; the ankle (4, 3.6%) for 1 infection; and the knee (7, 6.3%) for 1 infection. The Achilles region (3, 2.7%) and the remaining sites (forearm, shoulder, thigh, neck, back, chest) had no complications. All patients were followed up at 6–12 weeks, depending on clinic availability.

**Table 1:** Remote flap — table of complications. (Fragile wounds are not counted as complications but are included to show that sutures were left in place longer than the usual 7 days.)

Site	Total No.	Infection No.	Dehiscence No.	Fragile No.	Incomplete Excision
leg	78	2	1	3	2
forearm	8	0	0	0	0
Achilles	3	0	0	0	0
Ankle	4	1	0	2	0
Shoulder	5	0	0	0	0
Knee	7	1	0	3	0
Thigh	1	0	0	0	0
Neck	2	0	0	0	0
Back	3	0	0	0	0
Chest	1	0	0	0	0
**Total**	**112**	**4**	**1**	**8**	**2**

All 112 procedures in this consecutive series were remote flaps, performed as local or pedicled skin flaps; no free flaps were used for these cutaneous defects. These remote flaps represented only a small proportion of the author's total reconstructive workload over the study period; during the same period the author also performed approximately 100 major pedicled and free-flap reconstructions. In routine practice, conventional local flaps account for approximately 90–95% of flap reconstructions and remote flaps for the remaining 5–10%; perpendicular common-point closure is reserved for the uncommon situation in which a wound is almost closing or very tight, with concerns about tension. The remote flap is therefore an adjunct within the reconstructive decision-making tree rather than a replacement for it.

Fig. 1, Fig. 2 present the distribution of lesion type and anatomical site as bar charts. Figs. 3–5 illustrate the technique's conceptual basis, including the common point from which multiple flap options can be designed and the redirection of laxity vectors. Fig. 6 shows a remote flap raised in the flank for a large back melanoma excision; Fig. 7 shows the technique used over a cardiac pacemaker to offset the suture line and reduce local wound burden; Fig. 8 shows a large cheek defect closed while keeping scar off the neck.

## Discussion

In appropriately selected patients the technique achieved a flap-related complication profile consistent with other flap and reconstructive methods used in similar anatomical regions, supporting its safety in the hands of an experienced decision-maker. The audit showed a 4.5% flap-related complication rate (95% CI 1.5–10.1%) with 100% flap survival. These Figs. sit within, or below, published local-flap and reconstructive complication ranges for comparable sites.^15^^,^^16^ and are consistent with outcomes reported for keystone flaps in high-tension defects.^13^ These are descriptive comparisons with the literature rather than a controlled comparison and should be interpreted accordingly.

There was no robust correlation between complication rate and defect size (mean 2.3 ± 0.8 cm). A non-significant trend toward higher rates was observed in traditional high-tension areas (e.g., ankle), though wide confidence intervals from small subgroups preclude firm conclusions. Excision-related incomplete margins (1.8%) reflect lesion-excision planning and are independent of the flap technique. The single minor dehiscence occurred in a patient whose defect had been suitable for a skin graft; the patient declined a graft and accepted a remote flap with an appreciation of the increased risk, and the wound healed within one week of suture removal.

During design it is important to evaluate the distance from the remote flap to the defect and any intervening adhesion zones, as both reduce the efficiency with which laxity is transmitted along the chain. A useful analogy is a chain threaded through the supporting poles of a fence: unless the chain is released at each pole, its movement is stifled at each fixed point. Anchoring adhesions are therefore released where it is safe and beneficial to do so; where a design can avoid them, that design is preferred unless release confers a clear functional or aesthetic advantage.

A common point (Fig. 5), often within the same or an adjacent angiosome, can be converted into either a local flap or a remote flap. The author routinely uses this point to make the initial incision, assess laxity, and proceed to whichever option is most appropriate. In this series, whenever a flap was technically feasible it was completed as planned, and conversion to a conventional local flap remained readily available — a reassuring feature for surgeons adopting the technique. Where the defect nears closure, incising and perpendicularly closing the common point (Fig. 4) may avoid the need to raise the whole flap.

The large cheek defect (Fig. 8) illustrates the technique's value in an aesthetically sensitive area, allowing tension-free closure while keeping scar burden off the neck, in keeping with this patient's preference.^17^ She was reviewed at 6 months and 1 year with no scar revision and reported satisfaction, although a formal patient-reported outcome measure was not obtained. The pacemaker case (Fig. 7) shows how a remote flap can offset a suture line away from an implanted device and reduce local wound burden. These cases suggest the technique may help preserve peri-defect vascularity and improve scar placement.^7^^,^^8^^,^^14^; however, these potential benefits were not directly measured in this audit — no perfusion assessment, validated scar scale, or patient-reported outcome was collected — and they remain hypotheses to be tested rather than demonstrated advantages.

The author performs a large volume of reconstructive surgery each week, comprising free flaps, pedicled flaps, local flaps, and skin grafts, selected according to what best suits the individual patient. The remote flap is not appropriate for every defect and has not replaced the standard reconstructive ladder, which remains in far more frequent use; rather, it adds a valuable option to the decision-making process. In the author's practice it is used routinely, on a daily or at least weekly basis, in patients judged likely to benefit from it.

A note on terminology: because “flap” can imply a specific vascularised unit, the phrase “remote flap” has the potential to be misread. The term is retained because the technique is executed by raising a flap, but it is defined explicitly as a strategy for recruiting regional laxity, applicable across flap types, to avoid confusion with any single flap classification or with distant relaxing incisions.

Limitations include the retrospective design, probable selection bias toward remote flaps in favourable cases, single-surgeon experience limiting generalisability, and the absence of a comparator group and of objective perfusion, scar, and patient-reported outcome data. Prospective, controlled studies comparing remote and local flaps, incorporating perfusion imaging, validated scar assessment, and patient-reported outcomes, are needed to confirm the potential advantages described here.

## Conclusion

This single-surgeon series shows that the remote flap technique is a safe and reproducible option for selected high-tension cutaneous defects, with 100% flap survival and a flap-related complication profile consistent with local-flap reconstruction. Defined as a strategy for recruiting regional laxity — executable as a local, pedicled, or free flap — it is teachable and readily adopted, retains conversion to conventional options at any point, and may in appropriate cases avoid escalation up the reconstructive ladder. Prospective comparative study is warranted.

## Ethical & data custodian approval

Obtained as appropriate.

## Declaration of generative AI and AI-assisted technologies in the manuscript preparation process

During the preparation of this work, the author used Claude (Anthropic), a large language model, to assist with language editing and formatting. The author reviewed and edited the content as needed and takes full responsibility for the content of the published article.

## Funding

None.

## Declaration of competing interest

None declared.
